# Whole genome sequencing data used for surveillance of *Campylobacter* infections: detection of a large continuous outbreak, Denmark, 2019

**DOI:** 10.2807/1560-7917.ES.2021.26.22.2001396

**Published:** 2021-06-03

**Authors:** Katrine Grimstrup Joensen, Susanne Schjørring, Mette Rørbæk Gantzhorn, Camilla Thougaard Vester, Hans Linde Nielsen, Jørgen Harald Engberg, Hanne Marie Holt, Steen Ethelberg, Luise Müller, Gudrun Sandø, Eva Møller Nielsen

**Affiliations:** 1 Statens Serum Institut, Department of Bacteria, Parasites and Fungi, Copenhagen, Denmark; 2 Danish Veterinary and Food Administration, Glostrup, Denmark; 3 Department of Clinical Microbiology, Aalborg University Hospital, Aalborg, Denmark; 4 Department of Clinical Medicine, Aalborg University, Aalborg, Denmark; 5 Department of Clinical Microbiology, Zealand University Hospital, Slagelse, Denmark; 6 Department of Clinical Microbiology, Odense University Hospital, Odense, Denmark; 7 Statens Serum Institut, Department of Infectious Disease Epidemiology and Prevention, Copenhagen, Denmark

**Keywords:** Campylobacter, surveillance, whole genome sequencing, outbreak, one health

## Abstract

**Background:**

*Campylobacter* is one of the most frequent causes of bacterial gastroenteritis. *Campylobacter* outbreaks are rarely reported, which could be a reflection of a surveillance without routine molecular typing. We have previously shown that numerous small outbreak-like clusters can be detected when whole genome sequencing (WGS) data of clinical *Campylobacter* isolates was applied.

**Aim:**

Typing-based surveillance of *Campylobacter* infections was initiated in 2019 to enable detection of large clusters of clinical isolates and to match them to concurrent retail chicken isolates in order to react on ongoing outbreaks.

**Methods:**

We performed WGS continuously on isolates from cases (n = 701) and chicken meat (n = 164) throughout 2019. Core genome multilocus sequence typing was used to detect clusters of clinical isolates and match them to isolates from chicken meat.

**Results:**

Seventy-two clusters were detected, 58 small clusters (2–4 cases) and 14 large clusters (5–91 cases). One third of the clinical isolates matched isolates from chicken meat. One large cluster persisted throughout the whole year and represented 12% of all studied *Campylobacter* cases. This cluster type was detected in several chicken samples and was traced back to one slaughterhouse, where interventions were implemented to control the outbreak.

**Conclusion:**

Our WGS-based surveillance has contributed to an improved understanding of the dynamics of the occurrence of *Campylobacter* strains in chicken meat and the correlation to clusters of human cases.

## Introduction

Human campylobacteriosis is the most commonly reported zoonotic disease in Europe, with 246,571 reported cases in the European Union (EU) in 2018 [1]. *Campylobacter* infections are predominantly food-borne, with poultry as the primary source. However, other transmission routes are known, such as bathing, drinking contaminated water or direct contact with animals. In food samples, the highest occurrence of *Campylobacter* was detected in fresh chicken meat (37.5% of samples tested) [1]. In Denmark, we had 5,389 registered human cases in 2019 (incidence: 93/100,000 inhabitants) and 33% of conventional chicken meat samples were positive for *Campylobacter* at slaughter [2]. Of note, one third of the human infections diagnosed in Denmark are estimated to be travel-related [3].

Efforts to identify the specific source of *Campylobacter* infection in humans are rarely made in Denmark or other countries. Therefore, relevant information for targeted public health actions to prevent *Campylobacter* infections often does not exist. For decades, surveillance of other food-borne pathogens, especially *Salmonella* and *Listeria,* with high-discriminatory typing methods has proved to be a powerful tool for outbreak detection and investigations as well as for following trends and emergence of epidemic strains. Similar typing-based surveillance for *Campylobacter* has not been widely used and generally has not been very useful for the decision-making process on mitigating efforts by the public health and food safety authorities. The high diversity of *Campylobacter* isolates and the general assumption that most *Campylobacter* infections are sporadic are plausible explanations.

We have previously shown that, based on whole genome sequencing (WGS) data of *Campylobacter* isolates in 2015–17, we could identify numerous small outbreak-like clusters and, in many instances, genetically link them to concurrent animal and food isolates [4]. A large fraction of all 774 clinical isolates (27%) could be genetically linked to broilers or chicken meat, whereas only a few clinical isolates (2%) could be genetically linked to cattle isolates. A Danish case–control study conducted in the same period pointed at several food sources of campylobacteriosis among children and young adults, including consumption of chicken meat, minced beef, and fresh strawberries [5]. Therefore, in addition to sampling of chicken meat, the Danish Veterinary and Food Administration (DVFA) initiated sampling and analysis for *Campylobacter* in several other food sources that were identified by the case–control study to obtain knowledge on the impact of these sources.

In our 2015–17 study, a comparison of human isolates to food and animal isolates was done retrospectively and therefore no specific public health actions were taken. To evaluate the value of a prospective and continuous WGS-based surveillance system for *Campylobacter* in Denmark, we initiated WGS of isolates from human cases and retail food samples as well as the concurrent analysis of these cross-sector data. Here, we report the first year of surveillance (2019) and show that integrated WGS-based surveillance of *Campylobacter* in humans and food sources can identify correlations between the occurrence of specific strains in chicken meat and in human infections. The surveillance was also able to detect prolonged or reappearing outbreaks, which allows for specific interventions to control *Campylobacter* in the food production chain and thereby prevent human infections.

## Methods

### Surveillance

The overall design of the WGS-based surveillance of *Campylobacter* in 2019 aimed at obtaining clinical isolates from 10 to 15% of all campylobacteriosis cases (laboratory-confirmed cases of *Campylobacter* spp.) in Denmark. Our primary focus was on one geographical area, northern Jutland (North Denmark Region) but we also included isolates from two other geographically distant areas, Funen and Zealand. Concurrently, *Campylobacter* isolates obtained as part of the official DVFA food control programmes were sequenced and compared with the clinical isolates. Fresh meats (chicken and beef) were sampled at retail stores in northern Jutland and at distribution centres covering the major retail chains in Denmark; organic and free range broilers were also sampled at slaughterhouses. Since a large outbreak was detected in the spring of 2019, the planned collection of data for the surveillance was supplemented with additional data to investigate this outbreak.

The surveillance data were kept in a BioNumerics database (version 7.6, Applied Maths, Sint-Martens-Latem, Belgium). Case information on clinical isolates (date of diagnosis, age, sex, geographical region) was linked from the Register of Gastrointestinal Infections to this database. Information on isolates from food samples was added (sample ID, date of sampling and meat category). Sequencing, cluster analysis and cross-sector comparison of isolates was performed continuously over the year.

### Clinical isolates

Strains of *Campylobacter jejuni* and *Campylobacter coli* isolated from stools samples from Danish diarrhoeic cases by routine stool culture were collected and analysed from March through December 2019. The surveillance was designed in collaboration with three regional clinical microbiology laboratories, which cover three geographically widespread areas of Denmark. All isolates from patients diagnosed in the North Denmark Region covering northern Jutland (representing ca 10% of the national cases) were referred to the reference laboratory at Statens Serum Institut (SSI), as well as ca 10 isolates per month from the laboratories covering the areas of Funen and Region Zealand (the first 10 diagnosed cases per month in each area). A few clinical *Campylobacter* isolates from other regions were received at the reference laboratory at SSI during the study period and these were also included in the surveillance. Furthermore, available clinical isolates from end-October 2018 to February 2019 were analysed retrospectively as part of the outbreak investigation.

### Food samples

The DVFA sampled chilled chicken meat from retail stores in northern Jutland and at distribution centres covering the major retail chains in Denmark. Of note, while most chicken was of Danish origin, a few isolates were obtained from imported chicken meat. Samples of skin from leg quarters from organic and free range broilers were randomly sampled at slaughterhouses and samples of minced beef of Danish and non-Danish origin were sampled at retail level.

As part of the outbreak investigation, samples of different kinds of chilled chicken meat were obtained at one slaughterhouse. Batches containing meat from the identified farm were sampled.

*Campylobacter* was detected in chicken meat and skin from leg quarters by direct plating on Abeyta–Hunt–Bark agar (produced in-house) according to the standard method [6] with a detection limit of 10 colony-forming units/g. Minced beef samples were analysed using real-time PCR (BAX system real-time PCR assay for *Campylobacter*, Hygiena, Camarillo, CA, USA) after selective enrichment in Bolton Broth without blood (Oxoid, Roskilde, Denmark).

### Whole genome sequencing and cluster analysis

We performed WGS, quality control of WGS data, species identification, and comparison of WGS data by core genome multilocus sequence typing (cgMLST) as described in Joensen et al. [4]. Briefly, sequencing was conducted with Illumina technology (MiSeq or NextSeq sequencing machines, Illumina, San Diego, CA, USA) and the analysis was carried out using a BioNumerics database in which cgMLST was assigned using assembly-free and assembly-based allele calling, based on the 1,343 loci cgMLST scheme by Cody et al. [7]. In the present study, clusters of clinical isolates and matches to chicken isolates were defined on the basis of cgMLST allele differences using a cluster threshold of four with single-linkage clustering, instead of unweighted pair group method with arithmetic mean (UPGMA) clustering. Clusters ID were assigned as sequence type (ST) based on MLST followed by a number. Single nucleotide polymorphism (SNP) analysis was performed as an additional evaluation of the ST122#1 outbreak, using NASP [8] with default parameters with subsequent removal of recombination using CleanRecomb [9].

The data for this study have been deposited in the European Nucleotide Archive (ENA) at the European Molecular Biology Laboratory-European Bioinformatics Institute (EMBL-EBI) under accession number PRJEB41421 (https://www.ebi.ac.uk/ena/browser/view/PRJEB41421) and ERS4424632 (https://www.ebi.ac.uk/ena/browser/view/ERS4424632).

### Antimicrobial resistance

Genetic markers for acquired antimicrobial resistance were extracted based on the ResFinder database (https://cge.cbs.dtu.dk/services/ResFinder) [10] as part of our internal quality control pipeline. An in-house script was employed for detection of specific point mutations related to resistance to quinolones (nalidixic acid and ciprofloxacin): *gyrA* (A70T, D85Y, T86I, T86A, T86K, T86V, D90A, D90N, D90T, D90Y, P104S); erythromycin: *rplD* (G74D, G67V, A71D, R72I), *rplV* (G86E, A88E), and 23S (A2074G, A2074T, A2074C, A2075G); and streptomycin: *rpsL* (K43R, K88E, K88R, K88Q).

A random selection of six isolates of ST122#1 were subjected to antimicrobial susceptibility testing for six antimicrobial agents (ciprofloxacin, nalidixic acid, erythromycin, gentamicin, streptomycin, and tetracycline) as previously described [11]. Briefly, testing was performed as a minimum inhibitory concentration (MIC) determination using broth microdilution and interpreted by the use of European Committee on Antimicrobial Susceptibility Testing (EUCAST) epidemiological cut-off values [12].

## Ethical statement

No ethical approval was required for this study, since the data employed was obtained as part of our routine surveillance.

## Results

### Clinical isolates

In 2019, 5,389 cases of campylobacteriosis were registered in Denmark (93/100,000 inhabitants). This was a marked increase compared with the 4,546 cases in 2018 [2]. For the WGS-based surveillance, 626 clinical *C. jejuni* and *C. coli* isolates were continuously collected. In addition, 75 clinical isolates collected from the end of October 2018 through February 2019, were retrospectively included in the study as a follow-up on a detected outbreak. A total of 701 human clinical isolates of *C. jejuni* (n = 652; 93%) and *C. coli* (n = 49; 7%) were analysed. Of these, 453 isolates were from northern Jutland, 113 were from Funen, 111 were from Zealand, and 24 were from other laboratories in Denmark. This study represented 12.4% (668/5,389) of all reported cases in 2019 (Table).

**Table:** Clusters of *Campylobacter* spp. among human isolates and clinical clusters matching *Campylobacter* spp. from chicken, Denmark, October 2018–December 2019

Cluster size	All clinical clusters		Clinical clusters matching chicken isolates	
	**Clusters** **(n)**	**Human isolates (n)**	**Clusters ** **(n)**	**Human isolates (n)**
Large clusters (≥ 5 human cases)	14	200	11	179
Small clusters (2–4 human cases)	58	139	14	37
Sporadic cases with match	NA	3	3	3
Sporadic cases without match	NA	359	NA	NA

NA: Not applicable

### *Campylobacter* in food samples

*Campylobacter* was detected in 22% (196/909) of retail chicken meat samples. Of the total, 852 samples were Danish-produced (19% positive; 163/852) and 57 samples were of non-Danish origin (58% positive; 33/57). *Campylobacter* was detected in 68% (84/123) of skin samples from organic and free range broilers obtained at slaughterhouses. *Campylobacter* was not detected in any of the 402 samples of minced beef.

As part of the planned surveillance, 128 isolates of *C. jejuni* (n = 106; 83%) and *C. coli* (n = 22; 17%) from food samples were sequenced. Of the total, 52 isolates from chicken meat (45 from Danish-produced meat, seven of non-Danish origin), mainly from northern Jutland, and 76 isolates from organic and free range broilers.

As part of the outbreak investigation, 36 *C. jejuni* isolates from control samples obtained at the implicated slaughterhouse were sequenced and included in the analysis (see details below). These comprised 15 isolates from conventionally produced broilers, 11 isolates from samples of three batches containing meat from the implicated farm, and 10 isolates from two environmental samples from the slaughterhouse.

### Clusters of clinical isolates and matches to chicken isolates

Almost half of the clinical isolates (48%; 339/701) clustered with other clinical isolates: 200 were part of 14 large clusters (five to 91 cases) and 139 were part of 58 small clusters (two to four cases) (Table). All large clusters and the majority of the small clusters were caused by *C. jejuni,* while four small clusters caused by *C. coli* were detected.

One third of all clinical isolates from humans (31%; 219/701) matched chicken isolates. Most of these clinical isolates were part of the large clusters. Specifically, 179 clinical isolates represented the 11 large clusters with a detected source, while for three large clusters no source match was initially detected. Thirty-seven clinical isolates were associated with 14 of the 58 small clusters (24%), and only three sporadic human cases matched a chicken isolate. A single cluster (ST9882#1) of five clinical cases was matched to one of the seven isolates from imported chicken meat.

Of the 128 isolates obtained from the surveillance of chicken meat, 90 different cluster types (cgMLST) were detected and 35% (45/128) of the isolates could be genetically matched to clinical isolates. Isolates from conventional chicken meat (58%; 30/52) more often matched clinical isolates than those from organic chicken meat (20%; 15/76).

Of the three large clusters of clinical cases without a source match, one cluster (ST42#2) was confined to the island of Bornholm with milk as the suspected source. Another cluster (ST48#1) was a reappearance of a strain from a previous outbreak in 2016 with 17 cases that matched isolates from Danish-produced chicken from the same year. The third large cluster (ST257#5) without a chicken match was atypical; all five cases were from the same geographical area (northern Jutland) and appeared within 2 weeks in September 2019.

### Detection of a large prolonged outbreak

**Figure 1:**
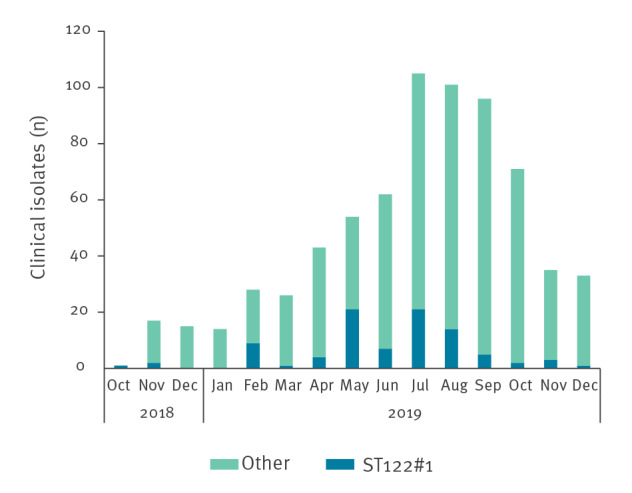
Number of sequenced clinical *Campylobacter* sequence type (ST)122#1 and isolates of all other types by month, Denmark, October 2018–December 2019 (n = 701)

The largest cluster was the ST122#1 cluster with a total of 91 cases. This cluster type was first detected in the beginning of the surveillance period, March 2019, and continued to be present during the whole year, peaking in the period May–August 2019. The retrospective analysis of isolates from the preceding months showed that this strain existed among human cases already in October 2018, and three isolates of ST122#1 were seen among the 33 sequenced isolates from late 2018. ST122#1 constituted 13% (88/668) of all clinical isolates in 2019 (Figure 1).

ST: sequence type.

The ST122#1 cluster included human cases with an age range of 2–91 years (median: 49 years) and a higher proportion of males (58%; 53/91) than females. Cases were from all geographical areas: 58 cases from northern Jutland, 19 cases from Funen, 12 cases from Zealand, with an additional two cases from other laboratories. Thus, the outbreak strain constituted an almost even proportion (11–17%) of the typed clinical isolates across the areas.

The ST122#1 cluster matched four chicken isolates obtained from the sampling of retail meat: two sampled in May 2019 and two in August 2019. These isolates were traced back to a single slaughterhouse. The slaughterhouse and the DVFA investigated the possible source, both at farm and slaughterhouse level, through extensive sampling and retrospective analysis of available isolates. Microbiological follow-up investigations revealed an additional 30 ST122#1 isolates: 20 isolates obtained from meat samples at the slaughterhouse between late February and the beginning of August 2019 and 10 isolates collected from the slaughterhouse environment in late October 2019. Based on the slaughter dates, these results supported the connection of the outbreak strain to one specific farm. From August 2019, the slaughterhouse decided that meat from this farm should be frozen to reduce the quantitative level of *Campylobacter*. Furthermore, the slaughterhouse outlined an action plan for optimising procedures and equipment. It is probable that other farms harboured the same strain. However, at this point in time, no other matches were found among the limited number of analysed samples.

**Figure 2:**
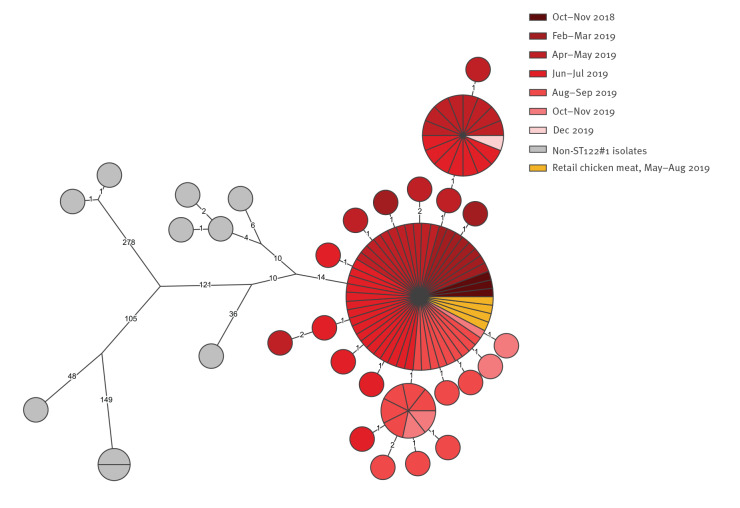
Phylogenetic (SNP) tree of sequence type (ST)122 isolates, including ST122#1 outbreak and non-related isolates, Denmark, October 2018–December 2019 (n = 105)

Although the ST122#1 cluster type persisted for more than a year, the genome seemed to be stable over time with 0–6 SNPs between any isolate (Figure 2). All ST122#1 isolates were within 0–5 allelic differences using cgMLST. Furthermore, the ST122#1 isolates were 28–427 SNPs (minimum 25 allelic differences) from other ST122 isolates in our database. The ST122#1 strain was found to be resistant to ciprofloxacin (MIC 8–16 µg/ml), nalidixic acid (MIC 64 µg/ml) and tetracycline (MIC 64 µg/ml), phenotypic traits facilitated by the presence of the *gyrA* T86I point mutation and the presence of the *tet(O/32/O)* gene. The strain was sensitive to erythromycin, gentamicin and streptomycin.

The maximum parsimony tree (logarithmic scaling) was constructed using an internal outbreak isolate as reference (ERS4424632). Colours indicate the retail chicken isolates and the month of sampling in 2018–19 for the human isolates of ST122#1.

## Discussion

We initiated a WGS-based surveillance of *Campylobacter* in Denmark in 2019, where we continuously compared a subset of clinical *Campylobacter* isolates from humans to isolates from the official sampling of chicken meat performed by DVFA. Our study has shown that roughly half of human cases belong to genetic clusters, that almost one third of all clinical isolates match a chicken source, and that most large clusters of human cases can be linked to chicken sources by WGS. These findings are in line with our previous study [4], but contrary to previous years, the surveillance in 2019 led to the detection of an unusually large genetic cluster that persisted throughout the year. While we do not have reliable travel information for all cases in the routine surveillance data, 30–36% of interviewed campylobacteriosis cases reported foreign travel in the 14 days before symptom onset in the years 2008–2015 [3]. Therefore, we can postulate that both the large outbreak and other cases whose isolates match those obtained from fresh chicken from the Danish market, make up an even larger proportion of the domestically-acquired infections than we estimated here.

The 2019 surveillance detected 88 clinical isolates of the outbreak cluster strain ST122#1. Since only a subset of the national isolates was typed, we estimate that the actual number of cases affected by this outbreak strain was ca 700 (13%) of all registered cases in 2019. Although the number of ST122#1 cases generally seemed to follow the typical seasonal variation for campylobacteriosis, there was a clear decline in the relative number of ST122#1 cases after August 2019. Potential explanations for the decline could be implementation of the general interventions at the implicated slaughterhouse and/or the fact that meat from the suspected farm was only distributed frozen after August 2019, although the contribution of each measure is uncertain. Freezing reduces the *Campylobacter* count in contaminated chicken meat and it is estimated that a significant public health risk reduction can be achieved by freezing chicken carcasses [13]. A few human cases with the outbreak strain were also seen in the beginning of 2020 (data not shown) and it is possible that it had spread to multiple farms and/or other slaughterhouses.

The persistence of this outbreak strain might be due to specific strain traits or factors related to the chicken production or possibly a combination of these. Further examination of this could be relevant for prevention of similar outbreaks in the future. For example, some strains might have the ability to colonise the chicken gut at higher levels [14-16], thereby increasing the risk of meat contaminated with high concentrations of the pathogen. Also, Wilson et al. [15] suggested that growth in the chicken gut increases the genetic diversity of subpopulations of *C. jejuni,* which possibly drives adaption to colonisation in humans. Several examples of newly emerging strains spreading within chicken production lines have been described; antimicrobial resistance of the strains has been mentioned as a possible factor explaining their persistence and spread of the strains. In the UK, the recent emergence of the ST5136 strain was suggested to result from its gradual acquisition of AMR genes in parallel with the high use of antibiotics in the poultry production [17]. In New Zealand, the ST6964 strain that emerged and rapidly spread in the poultry production was tetracycline- and quinolone-resistant [18], which was also the case for our ST122#1 outbreak strain. In the Danish poultry production, the use of fluoroquinolones is negligible, whereas tetracycline is one of the antimicrobials used for treatment of disease outbreaks in poultry; generally, there is a low consumption of antimicrobials in the Danish broiler production [19]. Interestingly, over the past few years, we have seen a marked increase in tetracycline resistance in clinical *C. jejuni* as well as chicken isolates in Denmark. In 2018, 32% of isolates from broilers slaughtered in Denmark and 34% of isolates from domestically-acquired human infections were resistant to tetracycline [19].

At present, experience with WGS-based analysis and interpretation of prolonged *Campylobacter* outbreaks is limited. Despite this, we found that the ST122#1 outbreak strain displayed little variation between isolates spanning the whole year, which argues for genomic stability and is in line with what is known for other food-borne pathogens [20,21]. Although ST122 is not one of the most prevalent STs in Denmark or abroad (ST122 constitutes 735 out of 98,830 of isolates in the PubMLST database at https://pubmlst.org/campylobacter; accessed: 9 November 2020), we have seen several examples of other ST122 strains in Denmark and find there is generally a large diversity within ST122 and outbreak strains can easily be defined.

In our WGS-based surveillance setup, epidemiological follow-up with either electronic questionnaires or interviews was performed only on cases from selected, large genetic clusters (data not shown). As isolates from most of these large clusters were already known to match chicken isolates, there was limited added value of this effort since we found that most cases had consumed chicken, which was also true for the control population in the Danish case–control study [6]. Instead, for future WGS-based surveillance, it would be more meaningful to follow up rapidly on cases who are part of large clusters lacking a source-match, as this could potentially aid in detection of non-poultry sources of infection. For instance, we found a seemingly local and possible point-source outbreak of five cases who were all infected with an ST257#5 strain within a 2-week period without any food match. However, it is clearly a challenge to be able to timely detect and react to small and time-restricted outbreaks when only a subset of isolates from reported cases are typed.

Our surveillance setup highlighted the value of implementing a WGS-based surveillance for detection of large prolonged outbreaks, as these types of outbreaks offer the possibility of intervention and potential prevention of human cases. Also, identification of reappearing cluster types offers a potential to facilitate an earlier intervention. However, with this type of WGS-based setup, follow-up on each genetic cluster of *Campylobacter* is not feasible because of the large number of clusters, which would require disproportionate resources, but also because of the time factor as most cluster types have already disappeared before it is possible to take action. The WGS-based surveillance in 2019 has, combined with our previous study in 2015–17, allowed us to gain knowledge on the larger picture of the *Campylobacter* epidemiology in Denmark, and the dynamic of clusters throughout the year. It also shows that WGS-based surveillance of human infections without the comparison to concurrent food isolates is of limited public health value. WGS-based surveillance should be further strengthened by the inclusion of isolates from other food or environmental sources. For other countries to implement a WGS-based surveillance, it would be favourable to employ such a setup for a year or two in order to get an overview of the situation in their country. It may not be necessary to perform such an extensive surveillance each year, after knowledge of the status has been established. In Denmark, we continued WGS-based surveillance in 2020 with some modifications. WGS was performed on isolates from retail food, representing all geographical regions. We have also attempted to have a more even representation of clinical isolates across the country; four clinical laboratories (located in four of the five regions in Denmark) submitted a subset of isolates proportional to the total number of cases.

Our study had several limitations. Only a fraction of all isolates from clinical cases and food samples in the official DVFA control programme underwent WGS. This is a clear limitation and is mainly a matter of resources. Access to *Campylobacter* isolates is also limited due to the increased use of non-culture-based diagnostics. To obtain more isolates, the reference laboratory might have to perform culturing of PCR-positive samples, which requires additional resources and is often unsuccessful because of delayed culturing. Only isolates from chicken meat were available for this study, but other food sources, as well as direct animal contact and the environment are also likely sources of infection.

## Conclusions

WGS-based surveillance of clinical cases and chicken samples improved our understanding of the occurrence and dynamics of *Campylobacter* strains in chicken meat and the correlation to clusters of human cases. Typically, some strains were only present in chicken meat and/or human cases in periods of a few weeks or months, whereas the occurrence of other strains fluctuated over time in a way that could be related to the production cycles in each chicken farm. The most remarkable example is the ST122#1 strain that was present in human cases and in chicken meat as well as the slaughterhouse for at least one year. The present surveillance was not designed to elucidate other major sources of infections and, although focus was on detection of large outbreaks, only chicken-related outbreaks were solved since traditional epidemiological follow-up was found to have limited value. A main impact of the 2019 surveillance is that the clear association of a substantial proportion of the human *Campylobacter* cases to the Danish chicken production has increased the poultry industry’s awareness. Although the poultry industry has been fighting *Campylobacter* for years, this study – and specifically the large outbreak – has led to extensive initiatives and investments targeting *Campylobacter* throughout the production chain. These interventions started shortly after the outbreak was apparent and are still in progress, which is essential for obtaining a pronounced decrease in the occurrence of *Campylobacter* in chicken meat. Furthermore, the follow-up investigations have led to new insights and raised several questions regarding the epidemiology of *Campylobacter* as well as on the characteristics of the specific outbreak strain, which should be addressed in future studies. Hopefully, this knowledge and awareness will lead to a decrease in the Danish chicken-associated human cases of campylobacteriosis in the coming years.

## References

[1] European Food Safety Authority and European Centre for Disease Prevention and Control (EFSA and ECDC). The European Union one health 2018 zoonoses report. EFSA J. 2019;17(12):e05926. 32626211 10.2903/j.efsa.2019.5926 PMC7055727

[2] Anonymous, 2020. Annual report on zoonoses in Denmark 2019. Kongens Lyngby: National Food Institute, Technical University of Denmark; 2019.

[3] Kuhn KG Nielsen EM Mølbak K Ethelberg S. Epidemiology of campylobacteriosis in Denmark 2000-2015. Zoonoses Public Health. 2018;65(1):59-66. 10.1111/zph.12367 28597535

[4] Joensen KG Kiil K Gantzhorn MR Nauerby B Engberg J Holt HM Whole-genome sequencing to detect numerous Campylobacter jejuni outbreaks and match patient isolates to sources, Denmark, 2015-2017. Emerg Infect Dis. 2020;26(3):523-32. 10.3201/eid2603.190947 32091364 PMC7045838

[5] Kuhn KG Nielsen EM Mølbak K Ethelberg S. Determinants of sporadic *Campylobacter* infections in Denmark: a nationwide case-control study among children and young adults. Clin Epidemiol. 2018;10:1695-707. 10.2147/CLEP.S177141 30538574 PMC6255050

[6] Nordic Committee on Food Analysis (NMKL). Thermotolerant Campylobacter. Detection, semi-quantitative and quantitative determination in foods and drinking water. NMKL no. 119, 3rd ed. Copenhagen: NMKL; 2007.

[7] Cody AJ Bray JE Jolley KA McCarthy ND Maiden MCJ. Core genome multilocus sequence typing scheme for stable, comparative analysis of Campylobacter jejuni and C. coli human disease isolates. J Clin Microbiol. 2017;55(7):2086-97. 10.1128/JCM.00080-17 28446571 PMC5483910

[8] Sahl JW Lemmer D Travis J Schupp JM Gillece JD Aziz M NASP: an accurate, rapid method for the identification of SNPs in WGS datasets that supports flexible input and output formats. Microb Genom. 2016;2(8):e000074. 10.1099/mgen.0.000074 28348869 PMC5320593

[9] Østerlund M, Kiil K. CleanRecomb, a quick tool for recombination detection in SNP based cluster analysis. bioRxiv. 2018;317131. 10.1101/317131

[10] Zankari E Hasman H Cosentino S Vestergaard M Rasmussen S Lund O Identification of acquired antimicrobial resistance genes. J Antimicrob Chemother. 2012;67(11):2640-4. 10.1093/jac/dks261 22782487 PMC3468078

[11] Dahl LG Joensen KG Østerlund MT Kiil K Nielsen EM. Prediction of antimicrobial resistance in clinical Campylobacter jejuni isolates from whole-genome sequencing data. Eur J Clin Microbiol Infect Dis. 2021; 40(4):673-82. 10.1007/s10096-020-04043-y 32974772 PMC7979593

[12] European Centre for Disease Prevention and Control (ECDC). EU protocol for harmonised monitoring of antimicrobial resistance in human *Salmonella* and *Campylobacter* isolates - June 2016. Stockholm: ECDC; 2016. Available from: https://www.ecdc.europa.eu/sites/default/files/media/en/publications/Publications/antimicrobial-resistance-Salmonella-Campylobacter-harmonised-monitoring.pdf

[13] EFSA Panel on Biological Hazards (BIOHAZ). Scientific opinion on Campylobacter in broiler meat production: control options and performance objectives and/or targets at different stages of the food chain. EFSA J. 2011;9(4):2105. 10.2903/j.efsa.2011.2105 PMC13129689 42078736

[14] Ringoir DD Korolik V. Colonisation phenotype and colonisation potential differences in Campylobacter jejuni strains in chickens before and after passage in vivo. Vet Microbiol. 2003;92(3):225-35. 10.1016/S0378-1135(02)00378-4 12523984

[15] Wilson DL Rathinam VAK Qi W Wick LM Landgraf J Bell JA Genetic diversity in Campylobacter jejuni is associated with differential colonization of broiler chickens and C57BL/6J IL10-deficient mice. Microbiology (Reading). 2010;156(Pt 7):2046-57. 10.1099/mic.0.035717-0 20360176 PMC3068676

[16] Pielsticker C Glünder G Rautenschlein S. Colonization properties of Campylobacter jejuni in chickens. Eur J Microbiol Immunol (Bp). 2012;2(1):61-5. 10.1556/EuJMI.2.2012.1.9 24611122 PMC3933991

[17] Lopes BS Strachan NJC Ramjee M Thomson A MacRae M Shaw S Nationwide stepwise emergence and evolution of multidrug-resistant Campylobacter jejuni sequence type 5136, United Kingdom. Emerg Infect Dis. 2019;25(7):1320-9. 10.3201/eid2507.181572 31211671 PMC6590748

[18] French NP Zhang J Carter GP Midwinter AC Biggs PJ Dyet K Genomic analysis of fluoroquinolone- and tetracycline-resistant *Campylobacter jejuni* sequence type 6964 in humans and poultry, New Zealand, 2014-2016. Emerg Infect Dis. 2019;25(12):2226-34. 10.3201/eid2512.190267 31742539 PMC6874264

[19] DANMAP 2018. Use of antimicrobial agents and occurrence of antimicrobial resistance in bacteria from food animals, food and humans in Denmark. ISSN 1600-2032. Copenhagen: Statens Serum Institut, National Food Institute, Technical University of Denmark; 2018. Available from: www.danmap.org

[20] Gillesberg Lassen S Ethelberg S Björkman JT Jensen T Sørensen G Kvistholm Jensen A Two listeria outbreaks caused by smoked fish consumption-using whole-genome sequencing for outbreak investigations. Clin Microbiol Infect. 2016;22(7):620-4. 10.1016/j.cmi.2016.04.017 27145209

[21] Pightling AW Pettengill JB Luo Y Baugher JD Rand H Strain E. Interpreting whole-genome sequence analyses of foodborne bacteria for regulatory applications and outbreak investigations. Front Microbiol. 2018;9:1482. 10.3389/fmicb.2018.01482 30042741 PMC6048267

